# Widespread Arterial Thrombosis after ChAdOx1 nCov-19 Vaccination

**DOI:** 10.1155/2022/6804456

**Published:** 2022-02-16

**Authors:** Giorgio Berlot, Ariella Tomasini, Cristina La Fata, Stefania Pintacuda, Sara Rigutti, Anna Falanga

**Affiliations:** ^1^University of Trieste, Department of Anesthesia and Intensive Care, Italy; ^2^University of Milano Bicocca, Department of Immunohematology and Transfusion Medicine, Italy

## Abstract

Vaccine-induced thrombotic thrombocytopenia is an uncommon complication of COVID-19 vaccines using adenovirus mRNA carriers and has been associated with thrombosis of the cerebral venous sinuses and portal system. We report a case of a 69-year-old woman admitted to the intensive care unit due to stroke caused by thrombosis of the right carotid artery 9 days after receiving the ChAdOx1 nCov-19 vaccine. Further investigations demonstrated multiple thrombi in the arterial tree in the absence of any venous involvement. The clinical course and the treatment are described and discussed.

## 1. Introduction

A worldwide anti-SARS-CoV-2 immunization campaign commenced at the end of 2020 using different vaccines that induce the production of an immunological response against the viral spike protein (Sp). To this aim, two different strategies are used: the first takes advantage of S-encoding mRNA carried inside the cells by lipid nanoparticles (BNT162B2, Pfizer-BioNTech; mRNA-1273, Moderna), while the other uses engineered adenoviruses (ChAdOx1 nCov-19, AstraZeneca; Ad.26.COV2.S, Johnson & Johnson), whose genes coding for replication have been disabled and replaced with others encoding the Sp. Although all these preparations can cause transient flu-like symptoms, adenovirus-based vaccines have been associated with extremely rare occurrence (<1 case/100,000 doses) of a syndrome resembling heparin-induced thrombocytopenia (HIT), appearing 7–14 days after the injection and whose main features are the reduction of the platelet count and the formation of venous thrombi in both common and uncommon sites, including the cerebral venous sinuses (CVS) and portal system [1, 2]. A cause–effect relationship with the vaccine has been hypothesized by some authors, who described this condition as vaccine-induced thrombotic thrombocytopenia (VITT) [3]. The production of IgG directed against the platelet factor 4 (PF4) is a feature shared by both HIT and VITT, but in the former, the trigger for the immune reaction is represented by the link between the PF4 and heparin molecule [4]. In the latter, the biological smoking gun has not been identified yet [1–3].

Here, we report the case of a patient who presented with life-threatening diffuse arterial thrombosis without the involvement of the venous system.

## 2. Case Description

According to the current policies, all medical records are freely available for review and/or research purposes provided that the data remain anonymous.

**Figure 1:**
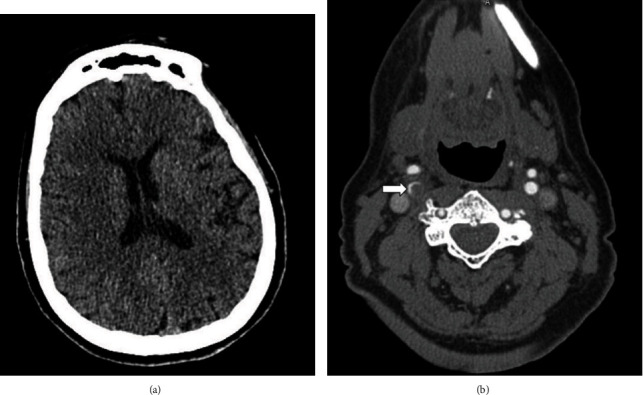
(a) Massive right hemispheric edema (admission); (b) occlusion of the right carotid artery (arrow).

A 69-year-old woman was admitted to the stroke unit due to left hemiparesis occurring 9 days after the first dose of ChAdOx1 nCov-19 vaccination. During this period, she complained of persistent headache initiated two days after the injection. Her medical history was negative apart from a hysterectomy performed 20 years prior to the current admission and arterial hypertension treated with valsartan. The first computed tomography (CT) angiography demonstrated massive ipsilateral hemispheric edema (Figure 1(a)) and the almost complete occlusion of the right internal carotid artery (ICA) and middle cerebral artery (MCA) (Figure 1(b)). A thromboaspiration under sedation was suspended after two hours because the removed thrombi were continuously replaced by newly formed ones, and the patient was then transferred to our intensive care unit (ICU). On admission, the patient was drowsy but arousable with verbal stimulation. The blood chemistries were entirely normal except for thrombocytopenia associated with reduced fibrinogen and elevated D-dimer levels (Table 1). The role of other prothrombotic factors, including antiphospholipid antibodies, hyperhomocysteinemia, and low ADAMTS-13 activity, was excluded. Suspecting a VITT, a second total body CT angiography was obtained that revealed multiple thrombi in the descending aorta, celiac tripod, inferior mesenteric artery, and minor branches of the left pulmonary artery in association with small ischemic areas in the left kidney, spleen, and liver, all in the absence of atherosclerotic plaques and venous thrombi. Pending the measurement of anti-PF4/heparin IgG, which was not immediately available in our hospital, the patient was with dexamethasone (40 mg IV for 4 days), intravenous immunoglobulins (IvIg) at a dose of 1 g/kg/day for 2 days, and IV argatroban titrated to maintain an activated partial thromboplastin time ratio of 1.5–1.8; intravenous 18% mannitol was administered to reduce cerebral edema. On the following day, the patient appeared drowsy and a brain CT demonstrated a right hemispheric infarction causing an initial brainstem compression. A decompressive craniotomy was performed following the suspension of argatroban. After the procedure, the patient recovered her consciousness but the left hemiparesis persisted. The argatroban infusion was restarted again 12 h after the intervention. The immunoenzymatic assay (PF4 Ig ELISA, Immucor GTI Diagnostics Inc.) performed in the hospital of Bergamo 2 days after admission demonstrated high levels of anti-PF4/heparin IgG (optical density units of 2630, whereas values > 1000 were considered strongly positive). In the following days, the clinical course was characterized by neurological stability and progressive increase in platelet count; repeated CT angiograms at 24 and 96 h did not detect any venous thrombosis. The administration of argatroban was discontinued after 7 days, and the patient was switched to oral dabigatran. Fourteen days after the ICU admission, the patient was transferred awake with a left hemiparesis to a neurorehabilitation unit. A CT scan angiography obtained 16 days after the ICU admission demonstrated the persistent occlusion of the right ICA whereas the right MCA territory was supported by leptomeningeal collateral activation; no venous thrombi were identified in the CVS or elsewhere.

## 3. Discussion

Although HIT and VITT share the same immunologic background, some relevant pathophysiological differences exist, as VITT also occurs in the absence of a previous or current heparin exposure and/or other causes of thrombocytopenia [5]. Different mechanisms have been hypothesized, including the following: (a) the prothrombotic properties of the adenoviral carrier that determines a complex interaction between the endothelium, leukocyte, and platelets, leading to their aggregation and subsequent vascular occlusion [6, 7]; (b) the effect of anti-S-Ig glycoprotein immunocomplexes that can activate the platelets by binding the FcɤRIIa causing their adhesion to the endothelial surface [8]. This reaction could be facilitated by the accidental entry of the vaccine into the bloodstream causing an elevated circulating viral load with the subsequent production of higher-than-expected amount of spike proteins. Since low amounts of PF4/heparin IgG can be found in approximately 8% of subjects observed in symptom-free patients receiving either mRNA or viral carrier vaccines [9], it is likely that a threshold level and/or other factors contribute to trigger the VITT. The diagnosis of VITT is based on a history of a recent vaccination against SARS-CoV-2 and thrombocytopenia causing thromboses mainly located in the venous circulation; actually, in studies involving hundreds of thousands of vaccinated subjects, the number of arterial events, occurring either isolately or in association with the former, is exceedingly uncommon [1] or even lower than expected in comparison with that reported in the years preceding the current pandemic [8].

All cases of VITT-related arterial stroke share some common features, including the time lag elapsing between the vaccination and the onset of symptoms, ranging between 7 and 10 days; the involvement of large extra and intracranial arteries; and the rapid neurological deterioration potentially leading to brain death: this clinical course has been described by De Michele et al. [10] and by Blauenfeldt et al. [11] in three patients who developed a massive hemispheric stroke following the thrombosis of the MCA that occurred between the 7^th^ and 9^th^ days following the vaccination with the ChAdOx1 nCov-19. In our case, despite an already established cerebral infarction in the same vascular territory of the patients described in these two studies [10, 11], the outcome was favorable; different factors account for this result, including the immediate suspension of heparin and its replacement with dabigatran, the immunomodulation with IvIg and steroids, and the wide decompressive craniotomy performed on the 2^nd^ day of ICU admission.

Thus, also in patients whose symptoms can be ascribed to arterial obstructions only, the rapid achievement of information about the vaccine status is warranted to initiate as soon as possible the appropriate treatment to prevent a possible catastrophic clinical course. Pending a laboratory confirmation that was unavailable at the time of ICU admission, we used this approach in our patient as the history and coagulative abnormalities prompted us to adopt the protocol indicated for the VITT-associated CVS [1, 2, 5, 8]. The dual goal of halting the production of anti-PF4/heparin IgG and preventing the extension of the already existing thrombi is accomplished with anticoagulants other than heparin, including fondaparinux and argatroban, possibly associated with IvIg or plasma exchange in nonresponders [14]. In our patient, the choice of argatroban was based on its better safety profile, and in a recent meta-analysis, its use was associated with a lower rate of hemorrhagic and thrombotic complications as compared with other anticoagulants [15]. Moreover, its short plasma half-life (<1 h) makes it suitable for subjects at risk of urgent surgical procedures, as occurred in our patient in whom the medical therapy failed to reduce the cerebral edema [16]. The measurement of anti-PF4/heparin IgG was not repeated as the platelet values rapidly increased after the initiation of the immunomodulating therapy.

## 4. Conclusion

Although CVS is considered a hallmark of VITT, its absence cannot exclude this diagnosis. As its successful treatment is time-dependent, a high index of suspicion and an aggressive approach even before the measurement of anti-PF4/heparin IgG are warranted in the presence of thrombi limited to the arterial tree occurring 1–2 weeks after the COVID-19 vaccination.

## Figures and Tables

**Table 1:** 

Variable	N.V.	D1^∗^	D2	D3	D4	D5	D6	D7^∗∗^	D10^∗∗∗^
aPTT ratio	0.76-1.18	1.13	1.55	1.76	1.83	1.96	1.77	1.80	1.87
PTr	0.78-1.20	0.91	1.61	2.37	1.77	1.79	1.80	2.09	2.11
Platelets (∗1000/ml)	150-450	55	56	77	92	102	125	150	230
D-dimer (ng/ml FEU)	<500	31.000	24.394	19.900	13.500	12.550	11.100	6.200	5941
AT III (%)	78-124	106	102	109	105	115	105	110	112
Fibrinogen (mg/dl)	160-380	150	144	174	224	300	360	360	700

Legend: N.V.: normal values; AT III: antithrombin III; x; ∗: before argatroban; ∗∗: argatroban discontinued; start dabigatran; ∗∗∗: 3 days after the initiation of dabigatran.
